# Supplementary material for: Association between Dietary Patterns Reflecting C-Reactive Protein and Metabolic Syndrome in the Chinese Population
Source: Nutrients. 2022 Jun 21;14(13):2566. doi: 10.3390/nu14132566 (PMC9268474; doi:10.3390/nu14132566)
Supplement: Supplementary file 1 [file nutrients-14-02566-s001.zip › nutrients-1778833-supplementary.pdf]

Supplemental Table S1. Sensitivity analysis on the associations between dietary pattern and metabolic syndrome (MetS).

	OR (95% CI)					P_{trend}
	Q1	Q2	Q3	Q4	Q5	
The main analysis						
MetS	1.00	1.10(1.02,1.19)	1.14(1.05,1.22)	1.23(1.15,1.33)	1.49(1.38,1.61)	<0.001
Abdominal obesity	1.00	1.11(1.05,1.18)	1.14(1.07,1.21)	1.22(1.15,1.29)	1.45(1.36,1.54)	<0.001
Hyperglycemia	1.00	1.16(1.07,1.25)	1.26(1.17,1.36)	1.35(1.25,1.46)	1.52(1.40,1.65)	<0.001
High blood pressure	1.00	1.09(1.02,1.16)	1.11(1.04,1.19)	1.11(1.04,1.18)	1.19(1.11,1.28)	<0.001
Hyperlipidemia	1.00	0.96(0.92,1.00)	0.95(0.91,0.99)	0.95(0.91,1.00)	1.00(0.96,1.06)	0.74
Additional adjust for occupation						
MetS	1.00	1.11(1.02,1.20)	1.20(1.11,1.29)	1.25(1.16,1.34)	1.50(1.39,1.62)	<0.001
Abdominal obesity	1.00	1.12(1.05,1.19)	1.17(1.11,1.25)	1.23(1.16,1.31)	1.44(1.36,1.53)	<0.001
Hyperglycemia	1.00	1.18(1.09,1.28)	1.28(1.19,1.38)	1.33(1.23,1.44)	1.52(1.40,1.64)	<0.001
High blood pressure	1.00	1.08(1.01,1.15)	1.12(1.05,1.20)	1.12(1.05,1.19)	1.19(1.11,1.27)	<0.001
Hyperlipidemia	1.00	0.95(0.91,0.99)	0.96(0.92,1.00)	0.95(0.90,0.99)	1.00(0.95,1.04)	0.68
Excluded missing value on sport						
MetS	1.00	1.10(1.00,1.20)	1.20(1.10,1.31)	1.24(1.14,1.35)	1.48(1.36,1.61)	<0.001
Abdominal obesity	1.00	1.09(1.02,1.17)	1.16(1.09,1.24)	1.24(1.16,1.32)	1.45(1.36,1.56)	<0.001
Hyperglycemia	1.00	1.18(1.08,1.29)	1.31(1.20,1.43)	1.32(1.21,1.44)	1.53(1.40,1.68)	<0.001
High blood pressure	1.00	1.08(1.00,1.16)	1.11(1.03,1.19)	1.11(1.03,1.19)	1.16(1.07,1.25)	<0.001
Hyperlipidemia	1.00	0.95(0.91,1.00)	0.97(0.92,1.02)	0.94(0.89,0.99)	0.99(0.94,1.05)	0.58

OR indicates odds ratio, Q indicates quintile. The results were based on model 2.

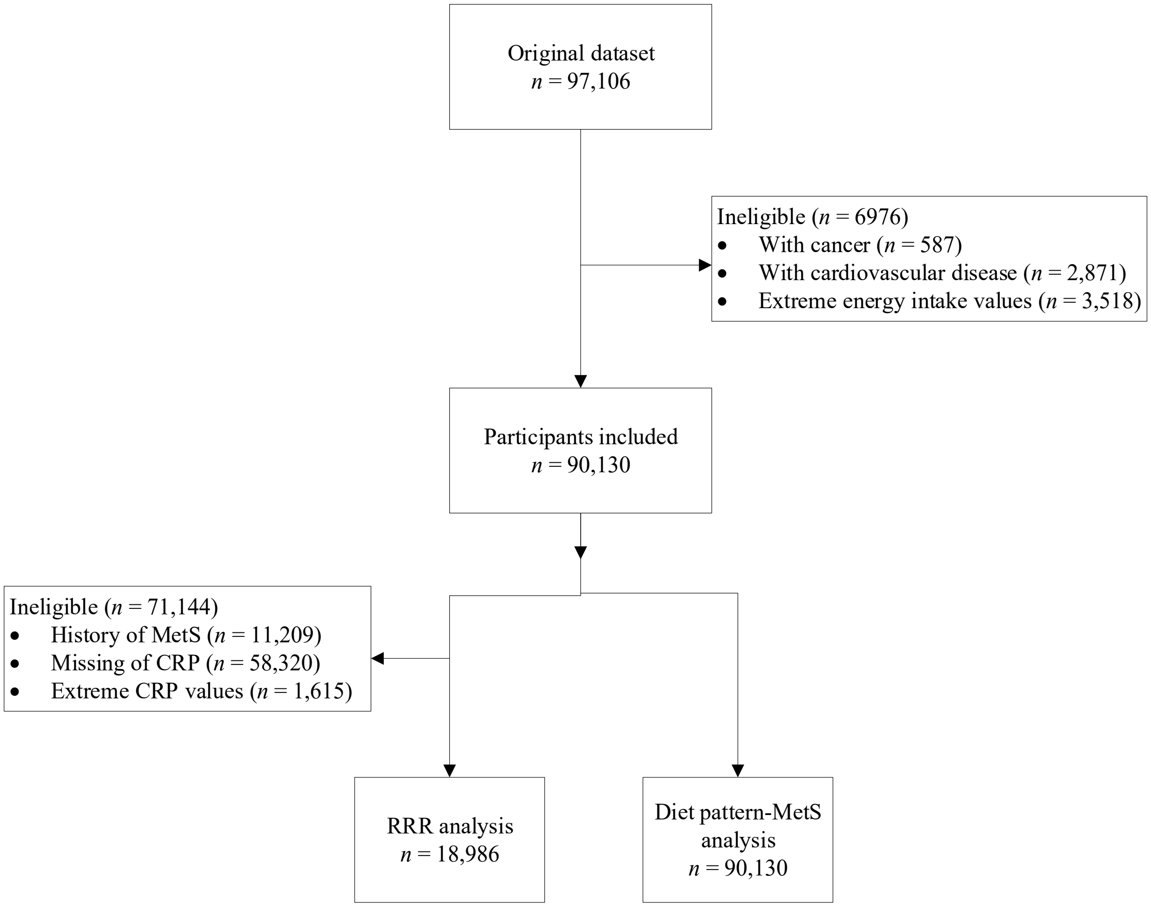

Supplemental Figure S1. Flow Chart for study population selection. RRR indicates reduced rank regression.

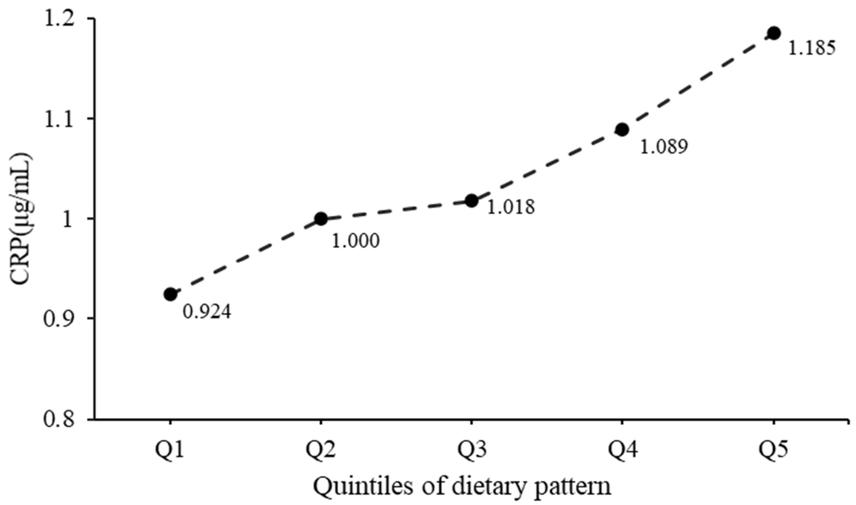

Supplemental Figure S2. C-reactive protein (CRP) values according to dietary patterns derived from reduced rank regression (RRR) in the Chinese population of MJ Health Database (*n* = 18,986). Q indicates quintile. Five groups were divided by the quintile of CRP-related dietary pattern score.
